# IscS Kinetics in Native Mass Spectrometry Buffers Reveal Key Physiochemical Properties that Influence Enzyme Activity

**DOI:** 10.1021/jasms.5c00280

**Published:** 2025-12-15

**Authors:** Shelby D. Oney-Hawthorne, David P. Barondeau, David H. Russell

**Affiliations:** Department of Chemistry, 14736 Texas A&M University, College Station, Texas 77842, United States

**Keywords:** native protein mass spectrometry, enzyme kinetics, volatile buffers, buffer capacity

## Abstract

Investigations of protein function and interactions with native mass spectrometry (MS) have yielded significant insights into protein dynamics, transient reaction intermediates, and pharmacokinetic targets. The pursuit of these studies and their outcomes depend on the preparation of protein samples in a manner able to support native conformation, active site chemistry, and protein–ligand interactions. Although ammonium acetate is a commonly utilized volatile buffer in MS-based analyses, the gap in buffer capacity near physiological pH calls into question whether this or other volatile buffer solutions are able to facilitate native conformation and protein–ligand interactions in the gas phase. We report enzymatic activity of the cysteine desulfurase IscS in four volatile buffer solutions comparable to that observed in traditionally utilized buffers such as Tris and HEPES, which is heavily influenced by buffer contributions to protein conformation and stability. We present a dual analysis of MS charge state and enzyme kinetics in the context of protein and solution physical properties, providing a chemical justification for the positive and negative effects of specific buffers. Ultimately, these results demonstrate how native MS technology can be used to identify protein conformational and dynamic interactions modulated by buffer systems to guide mechanistic studies.

## Graphical abstract

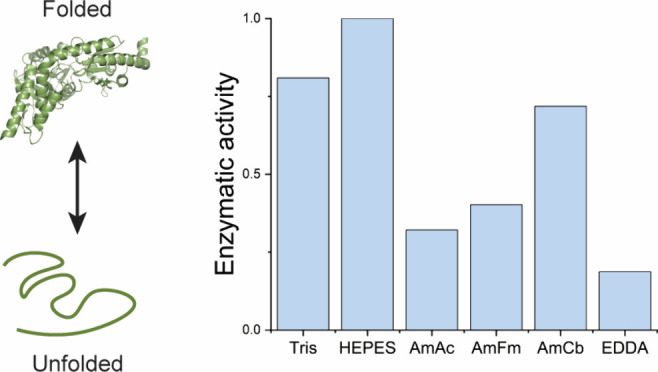

## Introduction

Native mass spectrometry (MS) is a powerful way to investigate protein topology, interrogate specific proteoforms, and probe protein–protein or protein–ligand binding kinetics.^1–6^ To provide physiologically relevant information, such bioanalytical MS applications require preserving noncovalent interactions that support native protein structure in the gas phase. Protein samples are usually prepared in volatile buffers that promote native electrostatic and hydrophobic interactions in solution and can be cleanly removed in the electrospray ionization (ESI) process, ideally producing adduct-free analyte ions for analysis.^7,8^ Typical volatile buffer systems for native MS studies, such as ammonium acetate, have a significant gap in buffering capacity near physiological pH 7.^9–11^ With limited or nonexistent buffer capacity, protein folding may no longer reflect native form. Although previous investigations reveal, in many cases, an agreement between experimental and computational data that indicate native protein structures and transient intermediates are maintained in the gas phase,^12–20^ this gap in buffering capacity may affect the type and extent of enzyme active site chemistry supported in MS-based investigations. Here, our objectives were to test and define the ability of a set of volatile MS buffers to facilitate native function and protein–protein interactions in the context of enzyme kinetic and gas-phase measurements.

Native MS is an emerging biophysical tool to probe native protein conformations along with protein–ligand, protein-nucleic acid, protein–lipid, and protein–protein interactions.^21,22^ Charge state analysis is often used to evaluate the extent of native-like protein conformation. Low charge states in narrow distributions tend to be consistent with a more native structure, and high and broad charge state distributions usually correspond to an unfolded state.^23,24^ Pastore and colleagues previously provided a practical guide for designing MS-based experiments to probe macromolecular interactions, concluding that while major advantages are associated with this type of investigation, cross-validation of results with other techniques is a wise precaution to ensure physiological significance.^25^

Buffer system selection for native MS experiments can significantly impact protein conformational state and reactivity in the gas phase. A study by Zenobi et al. highlighted the influence of native MS buffer identity on protein–ligand binding affinities, attributing these changes to charge reduction and evaporative cooling effects.^26^ Williams and co-workers identified structural inconsistencies in volatile and nonvolatile salt solutions by measuring temperature-dependent charge state effects of buffering species on protein conformation and stability.^27^ Haselberg and co-workers developed a direct size-exclusion chromatography to ESI-MS pipeline to test the agreement of solution protein conformations and gas-phase charge state distributions in different volatile buffers, noting signs of denaturation in chaotropic solutions such as ammonium formate.^28^ Russell and co-workers have reported charge reduction by alkyl ammonium acetate ESI buffers that also influence ligand binding (nucleotides) for both GroEL tetradecamer and the single ring GroEL mutant.^29–31^

The dimeric l-cysteine desulfurase (IscS) from *Escherichia coli* was selected as a model protein to evaluate the effects of volatile MS buffers on protein folding, protein–protein interactions, and activity. IscS is a member of the ISC pathway, a housekeeping Fe–S cluster biosynthetic system providing Fe–S clusters to several cellular pathways, and sulfur to additional target systems.^32–34^ IscS uses a pyridoxal phosphate (PLP) cofactor to generate a persulfide intermediate from its substrate l-cysteine and initiate Fe–S cluster biosynthesis in coordination with a scaffold protein IscU, a complex known for its metamorphic conformation states.^35–37^ Using IscS as a model protein, we intend to provide a realistic perspective on the activity of enzymes in volatile buffers for native MS relative to conventional buffers used in biochemical assays.

In this study, we probed the ability of four commonly used volatile buffers to support native-like properties for cysteine desulfurase activity. These results demonstrate the strengths and weaknesses of different volatile buffer options for facilitating chemistry involved in substrate turnover. Overall, our investigation of protein conformation and function in MS solutions has revealed key considerations for strategic sample preparation, including the physical properties of proteins and solution parameters such as pH and concentration.

## Experimental Section

### Expression and Purification of IscS

A pET vector carrying the *E. coli* IscS gene (Accession ID P0A6B7) with a kanamycin antibiotic resistance marker was transformed into BL21­(DE3) cells and expressed as described previously.^38^ In brief, cells were grown at 37 °C to OD_600_ 0.6, and expression was induced with 0.5 mM Isopropyl β-d-1-thiogalactopyranoside (IPTG). Growth was continued at 37 °C for 5 h, and then the cells were harvested by centrifugation. IscS was purified using a HisTrap HP Ni-NTA column and isolated by HiPrep 26/60 Sephacryl S-300 size exclusion chromatography (Cytiva). The concentration of IscS was determined by absorbance at 388 nm using an extinction coefficient of 6.6 mM^–1^ cm^–1^.

### Buffer Preparation

Ammonium acetate (AmAc), ammonium formate (AmFm), ammonium carbonate (AmCb), and ethylenediammonium diacetate (EDDA) buffers (MilliporeSigma) were prepared in 1 or 2 M stock solutions dissolved in LC-MS grade water, flash-frozen in liquid N_2_, and stored at −80 °C until use to prevent the evaporation of volatile ions (ammonia) over time. Just before preparing samples for analysis, aliquots were thawed, diluted to the desired working concentration (200 mM except where otherwise noted), and pH-adjusted to pH 8.5 using a 28% ammonium hydroxide solution (MilliporeSigma). This pH value was selected based on the activity profile of IscS, which showed optimum enzymatic activity at pH 8.5. Additionally, IscS remained stable for ESI-MS experiments in solutions at pH 8.5 but began to precipitate after a shorter period of time at pH 7.0. For these reasons, pH 8.5 was selected for the experiments presented.

Tris and HEPES buffers were freshly prepared with 50 mM Tris­(hydroxymethyl) aminomethane HCl or N-(2-hydroxyethyl) piperazine N’-(2-ethanesulfonic acid) and 150 mM sodium chloride (Research Products International) and pH-adjusted using a 6 M stock of sodium hydroxide (VWR International).

### Sulfide Detection Assay for Measuring Enzymatic Activity

Methylene blue assays were conducted in an anaerobic glovebox (≤0.4 ppm of O_2_) to determine IscS activity. Samples (800 μL total volume) of IscS (0.5 μM) and IscU (1.5 μM) were incubated with dithiothreitol (DTT, 4 mM) and ferrous acetate (0 or 250 μM) for 15 min at 37 °C. The cysteine desulfurase reaction was initiated as previously described^39^ with the addition of l-cysteine (1 mM) and quenched at 6 min with 200 μL of a 1:1 mixture of N,N-dimethyl-p-phenylenediamine (DMPD, 20 mM) and ferric chloride (30 mM). Samples were incubated for 20 min at 37 °C and then centrifuged for 5 min (15,000 rcf) to remove precipitated protein. Absorbance was measured at 670 nm and converted to a sulfide concentration based on a sodium sulfide standard curve.

### Native Mass Spectrometry Analysis

IscS samples were buffer exchanged into 200 mM volatile buffer solutions as indicated using pre-equilibrated Micro Bio-Spin P-6Gel Columns (Bio-Rad). Protein concentration was determined via UV–vis absorbance and adjusted to 1 or 2 μM before loading into pulled borosilicate emitter tips (Sutter Instruments). Samples were analyzed within 1 h of buffer exchange. Data sets were collected on a Q Exactive UHMR (ultrahigh mass range) Hybrid Quadrupole mass spectrometer (Thermo Scientific). Instrument parameters were optimized to minimize collisional activation while maintaining the best possible signal-to-noise ratio for each protein analyzed. The mass range was set to *m*/*z* 500–12000, capillary voltage 1.5 kV, CID 45.0 eV, CE 75.0, and capillary temperature 200 °C. Mass spectra were analyzed using UniDec^40^ to determine charge states and experimental masses.

## Results

The behavior of proteins in solution may vary extensively with the specific buffer in which the sample is prepared. Parameters such as solution pH, buffer capacity, and protein surface hydration in cooperation with buffer ions play a role for retaining native protein structure of the gas phase ions.^41–43^ Here, we determined changes in charge state and enzymatic activities for IscS in three volatile buffer solutions often used in native MS sample analysis: AmAc, AmFm, and AmCb. Additionally, a less common solution, EDDA, has been included based on successful use in previous investigations of the chaperone protein GroEL.^29,30,44^

The different volatile buffer solutions at pH 8.5 resulted in variations in the relative abundance of charge states and their distributions for each protein (Figure 1, Table S1). This pH value was selected to be consistent with the pH at which IscS demonstrates optimal activity. Although AmAc and AmFm solutions had generally consistent charge state distributions (Figure 1A and B), the most abundant forms were shifted toward lower charge states in AmFm solutions.

**Figure 1:**
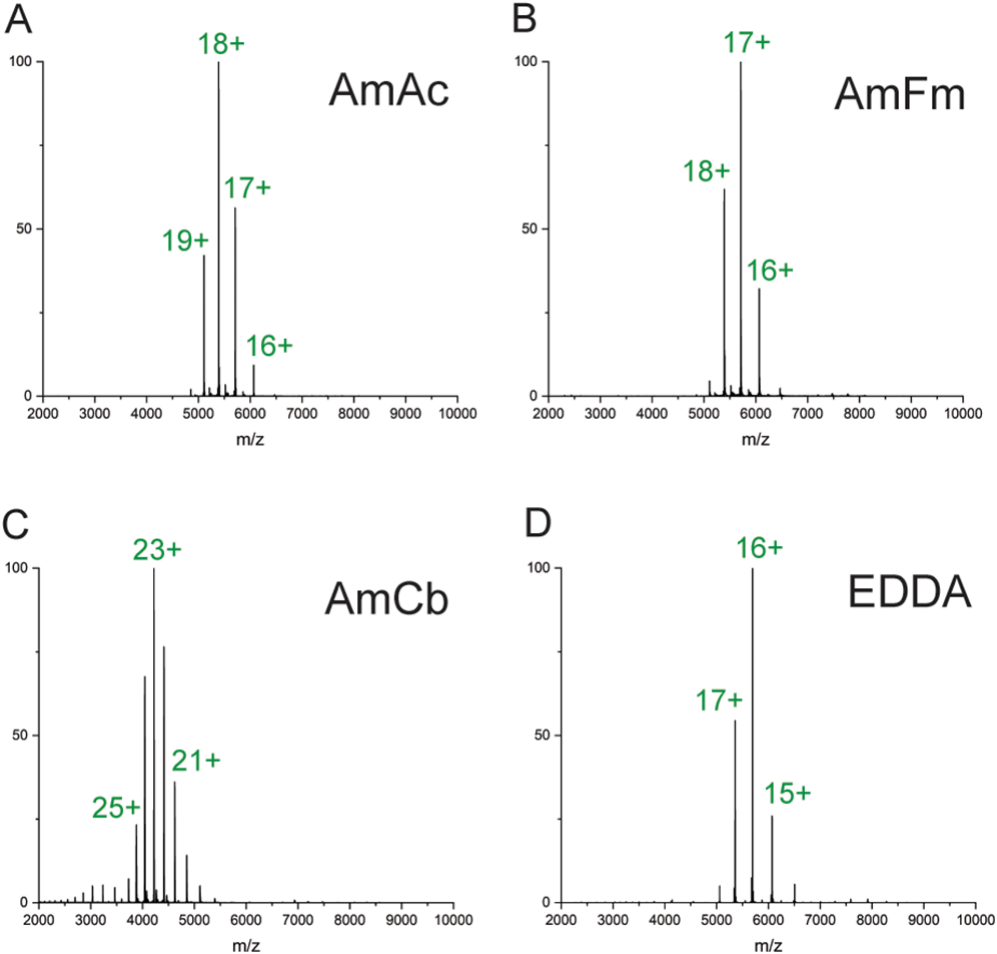
Mass spectra of IscS in (A) ammonium acetate, (B) ammonium formate, (C) ammonium bicarbonate, and (D) ethylenediammonium diacetate. Protein samples were buffer-exchanged into the solution indicated and diluted to 1 or 2 μM for analysis.

AmCb generated noticeably higher charge states with a much broader tailing pattern than those observed in the other volatile buffer solutions (Figure 1C), with an extended high charge state distribution indicative of unfolded species. This effect of AmCb has been previously documented and attributed to the outgassing of CO_2_, which causes varied degrees of unfolding during the electrospray process.^45^ In contrast, EDDA gave rise to narrow charge-reduced distributions (Figure 1D). These charge states represent multiple-protonated species formed on basic residues whose accessibility depends on the protein conformation.^23,46,47^ By this metric, higher charge states are typically associated with a more unfolded protein sample.^24^ The variable charge states observed are consistent with distinct shifts in protein conformations sampled in the different volatile buffer solutions. Protein restructuring in response to buffer ion interactions may also influence protein functionality. With this in mind, volatile buffer effects on the enzymatic activity of IscS were explored as a metric of native-like character in the context of mass spectrometry-based investigations

### Buffer Capacity Directly Influences Protein Performance and Is Controlled by Solution pH

An important factor to consider is the buffer capacity of the ion pair comprising a volatile buffer solution. This parameter is defined as the ability imparted by buffer ions to the solution to resist changes in pH, and it depends on the p*K*_a_ values of the buffering species.^48,49^ Buffering capacity sharply diminishes as the solution pH deviates from the p*K*_a_ of each buffer ion and is only an effective buffer when the pH is within ± 1 unit of the p*K*_a_ values.^9,50–52^ Notably, ammonium-containing (p*K*_a_ = 9.25) buffer solutions commonly used for native mass spectrometry offer minimal buffering capacity at physiologically relevant pH values near 7 (Table S1).

To probe the extent of native-like ligand binding chemistry critical for enzymatic activity in typical native MS sample conditions, we used a sulfide detection assay to quantify the enzymatic activity of IscS in pH-adjusted volatile buffer solutions. We selected buffer systems containing standard Tris^53^ or HEPES with NaCl as benchmarks for quantifying the extent of native-like cysteine desulfurase activity and for comparison to the volatile buffer systems AmAc, AmFm, AmCb, and EDDA. Activity measurements were determined for all solutions at pH 8.5 (within ∼ 1 pH unit of the p*K*_a_ values) for IscS alone and with the [2Fe-2S] scaffold protein IscU (Figure 2). Surprisingly, cysteine desulfurase activities measured in AmCb were consistent with values determined with the Tris/NaCl buffer system. The gap separating carbonate and ammonium buffering ranges is much smaller than those of the AmAc and AmFm ion pairs and may account for the more native-like activity in AmCb. In contrast, the IscS activity reported for EDDA was significantly lower than for AmCb despite having a similar gap in ion pair buffering capacities.

**Figure 2:**
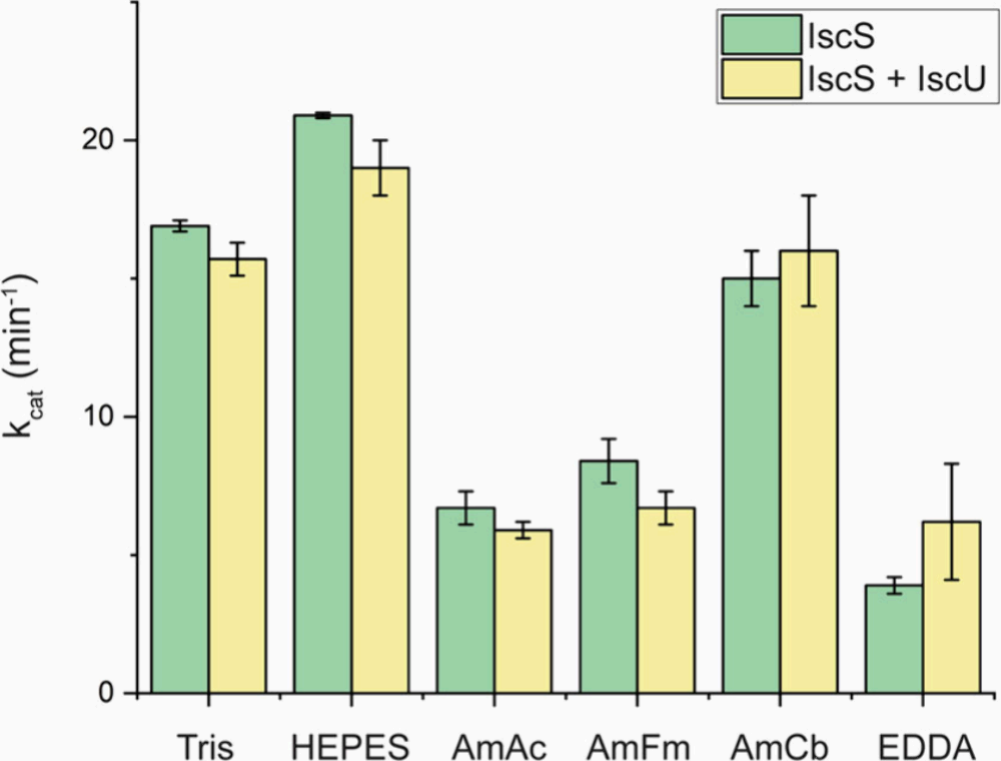
MS volatile buffers had a significant effect on IscS enzymatic activity. The rates of cysteine desulfurase activity of IscS with and without IscU were determined in four volatile buffers (200 mM each) and compared to 50 mM Tris/150 mM NaCl and 50 mM HEPES/150 mM NaCl controls. Sulfide production was quantitated at different l-cysteine concentrations to determine the *k*_cat_ values. All reaction mixtures were prepared at pH 8.5.

Knowing that buffer capacity is largest at pH values nearest the p*K*_a_ of buffering species, we hypothesized that adjusting the solution pH for each of the volatile buffer ions in the four MS solutions studied might increase IscS activity with enhanced buffer capacity. IscS performed optimally at the highest pH value tested (pH 8.5) near the upper p*K*_a_ values of ammonia and fully deprotonated EDDA (p*K*_a_ 9.25 and 9.46, respectively; Table S1, Figure S1). Conversely, activity measurements at pH values near the lower p*K*_a_ values of acetate, formate, carbonate, and singly deprotonated EDDA (p*K*_a_ 4.75, 3.75, 6.35, 6.42, respectively; Table S1) were undetectable except for AmCb (Figure S1C), which maintained approximately 70% activity relative to the pH 7.5 data set. In this case, pH adjustment to 7.0 resulted in the off-gassing of carbonate, a previously documented result of acidification from the initial pH of 8.3 AmCb as dissolved in water.^45^ It is likely that this effect generated a disproportionate amount of ammonium remaining in the solution that could contribute buffer capacity to support enzyme activity, which also accounts for the failure of EDDA to do so with a comparably lower p*K*_a_ (Figure S1D). For the other low-pH data sets, we suspect that the main reason for low or undetectable activity is due to high IscS active site protonation preventing persulfide chemistry from proceeding.

### The Buffer Capacity of Ammonium Acetate Does Not Depend on Concentration

Ammonium acetate is one of the most common buffers currently being used in laboratories for native mass spectrometry analysis, and we report moderate activity for IscS in this solution. To determine the potential role of ionic strength imparted by AmAc, we also evaluated the changes in enzymatic activity of IscS with AmAc concentration at pH 7.5. IscS exhibited about 27% of the activity in 200 mM AmAc relative to the Tris/NaCl data set (Figure S2, Table S3). There was a slight increase in observed activity at 100 mM AmAc to 32% relative activity, which then decreased to 28% in 10 mM AmAc and 16% in pure water. The increase in activity observed by decreasing AmAc concentration from 200 to 100 mM may be attributed to changing the ionic strength of the solution with decreasing buffer ion concentration.

Although often regarded as a positive factor for supporting protein stability, ionic strength trends inversely with the activity coefficient of ions in solution.^54^ The activity coefficient, which is the ratio of effective to actual ion concentration, represents a thermodynamic property that could account for deviation from ideal behavior.^55^ An ideal solution has an activity coefficient of one, and is defined as a solution with zero enthalpy of mixing (ΔH_solution_ = 0).^56^ Using the Davies equation derived from Debye–Hückel theory,^57^ it was determined that decreasing the AmAc concentration from 200 mM resulted in increasing activity coefficient values approaching one (Table S3). Diminishing enthalpic contributions from the buffering species may account for the increased activity observed at 100 mM AmAc before dropping again with further decreasing buffer concentration to mediate essential electrostatic interactions required for enzymatic activity. Overall, these changes in substrate turnover rate with AmAc concentration were minor and indicate that the buffer concentration can be tuned in this case study but is not sensitive to small changes.

## Discussion

In this study, we have reported solution and gas-phase data on the enzymatic activities and conformation states observed in protein samples prepared in four volatile buffers used in native MS analyses. For many proteins, these volatile buffer options could likely be used interchangeably for the observation of masses and simple analyses. With respect to more nuanced investigations into enzymatic activity and mechanistic steps of physiological reactions, however, we have demonstrated here that buffer selection can feasibly determine the outcome of a native MS experiment. For example, AmAc and AmFm are buffers of high molecular similarity that do not necessarily produce consistent results. Independent mass spectrometry-based studies on the mechanistic steps of [2Fe-2S] cluster biosynthesis have reported models for the initial steps of the reaction, with opposite conclusions derived from AmAc and AmFm data.^58,59^ Different protein folding and the resulting variable charge states observed in AmAc and AmFm may ultimately be most heavily influenced by a shift in droplet pH during the ESI process. A study by Konermann and colleagues on ESI droplet chemistry in ammonium acetate previously reported that the rapid evaporation of ammonia relative to acetate/acetic acid resulted in an approximately 1.6 pH unit drop from the initial pH.^10^ The p*K*_a_ of formate/formic acid is a full unit lower than that of acetate/acetic acid, and would likely result in a more extensive pH drop that would affect proteins differently on a case-by-case basis. Previously reported protein thermodynamics studies also suggest that the observed differences between buffers may also reflect small changes in the distribution of protein microstates.^29–31^

Although several factors may influence the response of a given protein in a buffer for native MS, an important consideration is buffer pH. Based on the pI of the protein, solution pH determines the protonation of surface-accessible residues that may be essential for mediating protein–substrate or protein–protein interactions. The consistently higher cysteine desulfurase activity of IscS observed in all buffers at pH 8.5 is likely a result of enhanced deprotonation of the sulfhydryl on the catalytic cysteine, which has a predicted p*K*_a_ of 8.0–8.5.^60,61^ Increased availability of the persulfide would enable more rapid substrate binding and turnover, which may result in the higher observed activities. This is supported by the low pH data sets, where the neutral or low net negative charge on the protein even near the lower p*K*_a_ buffering range failed to facilitate activity. Additionally, we note that the isoelectric point of IscS (pI = 6.03) is much lower than pH 8.5. From this, it can also be inferred that the net negative charge on IscS may be favorable for proper protein folding and surface hydration, which are required for native enzyme function in these samples. Based on the pH requirement to facilitate persulfide chemistry, it is clear that the buffer capacity of ammonium (p*K*_a_ 9.25) is critical for the success of an experiment or native measurement for this particular enzyme.

Kinetic trends in the IscS case study revealed that although Tris and HEPES with NaCl tended to facilitate the strongest enzymatic performance, AmCb was a strong contender in supporting cysteine desulfurase activity, producing thought-provoking results in solution that highlight how the extent to which buffers support protein function in solution may be different from how they affect the transition to the gas phase. The strong solution activity may be attributed to the more physiologically relevant buffering range of AmCb relative to AmAc and AmFm, making it more suitable for facilitating the reaction or possibly an advantage afforded by this buffer solution for allowing greater dynamic flexibility in solution. Conversely, cysteine desulfurase activity in EDDA was the lowest observed. We propose that this variable behavior of IscS can be explained in terms of the ability of the buffer to support active site chemistry, protein-solution ion interactions surrounding the metal/cluster binding site, and the extent of permitted protein flexibility. In the case of IscS, solution pH appears to affect the persulfide chemistry required for activity greatly. There are also metal coordination centers on IscS and IscU that are solvent-accessible and subject to the influence of buffer ions. The effects of buffer ion interactions with the metal centers of metalloproteins have been previously reported for Mn^2+^-dependent dioxygenase BLC230 and Fe^3+^-dependent catechol dioxygenase R01,2-CTD, where metal ion dissociation constants and corresponding enzymatic activities were determined in solutions optimized with respect to temperature and pH to demonstrate relative buffer effects on enzyme performance.^62^ These results revealed that the catalytic activities of the metalloenzymes studied were modulated directly by the influence of the buffer solution on enzyme metal affinity. Distinctive buffer effects on Fe^3+^-dependent R01,2-CTD were highlighted in the enzyme kinetic profile, showing the specific activity in Tris was almost twice that observed in HEPES but with a substantially higher K_m_, indicating decreased substrate affinity in Tris likely due to its strong metal-chelating properties. In support of this proposed metal-based buffer effect, the activity of nonmetalloenzyme trypsin remained unchanged with each of the buffers evaluated.

Buffer molecules may also affect enzymatic activity by acting as an inhibitor, in terms of ionic strength, and by direct modulation of structural characteristics and solubility.^63–66^ Ionic strength is known to directly influence protein behavior, with examples in literature where ionic strength has been used to control the activities and interfacial behavior of proteins through the dissociation and hydration of ions at the interfaces.^65–67^ As a common practical application, modulation of ionic strength is routinely used to support protein solubility or the intentional salting out of proteins, as in the ammonium sulfate precipitation method. In this technique, the hydration layer surrounding a protein is strategically disrupted by high concentrations of ammonium sulfate, leading to precipitation and easy separation of the desired protein from a heterogeneous sample.^68^ Protein solubility and specific pairwise interactions between small molecules and proteins depend on polar and charged residue side chains and bound ions that form short-range interactions such as salt bridges and hydrogen bonds to overcome conformational entropy opposing native protein folding.^64,69^ The electrostatic stability of proteins also depends on the hydration and adsorption of buffer ions to their solvent-accessible surfaces.^42^

Native protein structure is also determined by hydrophobic residues in the structure, conformational entropy, and steric factors.^70,71^ During translation, hydrophobic residues can be exposed and lead to detrimental misfolding.^72^ In the cell, chaperones mitigate folding errors by providing an environment apart from the crowded cytosol for the stabilization of non-native proteins.^73^ Also important are solvent-exposed hydrophobic regions, which accommodate protein–protein binding interactions.^74^ This makes striking the correct balance of relative solution polarity a requirement for maintaining protein folding (i.e., solubility) and binding events. The relative hydrophobic character of a protein can be quantified using the grand average of hydropathicity index (GRAVY), where positive and negative values indicate hydrophobicity and hydrophilicity, respectively.^75^ Using this metric, the calculated GRAVY value for IscS indicated predominantly hydrophilic character.

Given the variability of buffer effects observed, it is apparent that the identity of the ammonium counterion is extremely important to native protein conformation and function. Ultimately, this data demonstrates that although native-like character is achievable in MS buffers, using low and narrow charge state distributions as a sole metric for assigning native-like character is insufficient for enzymatic functions.

## Conclusion

Here we have presented an analysis of the effects of four native MS buffer solutions with respect to the physical properties of both the solutions and a model enzyme. Through this approach we demonstrated the variability of charge states and their distributions with different volatile buffers. The enzymatic activity of IscS was evaluated under sample preparation conditions relative to traditionally utilized biophysical assay buffers and it was determined that the type of active site chemistry is heavily influenced by the buffering species present.

From this study we draw two major conclusions. First, we report that native-like enzymatic activity comparable to that observed in traditional biophysical assay buffers such as Tris and HEPES is attainable in volatile buffers appropriate for MS studies. The second is a point of caution that a well-resolved ESI mass spectrum alone is insufficient evidence to conclude that an enzyme is being reported in its native form. For best results in studying dynamic protein systems and enzyme mechanisms, volatile buffers for MS should be selected on a case-by-case basis within the context of the physical properties of the protein, and parameters such as pH and concentration should be thoroughly optimized. This applies not only to MS-based investigations, but in fact all enzyme-based studies. Whenever possible, enzymes should be thoroughly characterized in the MS buffer to determine suitability. Although there is no universal volatile buffer, the options available afford opportunities to capitalize on the most desirable qualities for specific protein investigations. In addition to the buffers described in this study, alkyl ammonium acetate ESI buffers are emerging alternatives for protein studies, and success has also been recently reported with volatile buffers such as 2,2-difluoroethylamine (DFEA) and 2,2,2-trifluoroethylamine (TFEA) with more physiologically relevant buffering ranges for native protein MS.^29–31,76^ Taken together, these principles can be applied to improve strategies for sample preparation that improve or altogether change the outcomes of native MS protein investigations.

## Supplementary Material



## Notes

Physical properties of MS buffers and IscS, enzymatic activity variability with ionic strength and in response to changing pH, and protein purification procedures (PDF)
