# Supplementary material for: IscS Kinetics in Native Mass Spectrometry Buffers Reveal Key Physiochemical Properties that Influence Enzyme Activity
Source: J Am Soc Mass Spectrom. 2025 Dec 15;37(1):163–9. doi: 10.1021/jasms.5c00280 (PMC12784406; doi:10.1021/jasms.5c00280)
Supplement: Supplementary file 1 [file js5c00280_si_001.pdf]

Supporting Information

IscS Kinetics in Native Mass Spectrometry Buffers Reveal Key Physiochemical Properties that Influence Enzyme Activity

Shelby D. Oney-Hawthorne, David P. Barondeau, David H. Russell*

Department of Chemistry, Texas A&M University, College Station, Texas 77842, United States

*Email: russell@chem.tamu.edu

Table of Contents | Page

Table of Contents	Page
Table S1	S2
Table S2	S2
Table S3	S2
Figure S1	S3
Figure S2	S4
Protein Expressiion and Purification	S5
References	S5

Table S1. Physical properties of buffers at 25°C

	Low pK_a	High pK_a	Concentration (mM)	Ionic strength	Avg Charge State
**Tris/NaCl**	8.06		50/150	0.25	-
**HEPES/NaCl**	7.48		50/150	0.2	-
**AmAc**	4.75	9.25	200	0.2	18.6 ± 0.2
**AmFm**	3.75	9.25	200	0.2	16.6 ± 0.2
**AmCb**	6.35	9.25	200	0.6	20.8 ± 0.1
**EDDA**	6.42	9.46	200	0.6	15.83 ± 0.09

Table S2. Physical properties of proteins

	Molecular weight (kDa)	Oligomeric state	pI	GRAVY[1]	Aliphatic index[2]
**IscS**	97.1	Dimer	6.0	-0.327	87.38

Table S3. Calculated activity coefficient shifts with ionic strength

Ionic strength (mol kg^{-1})	Mean activity coefficient ($\gamma_\pm$)	k_{cat} (min^{-1})
**0.2**	0.75	2.9 ± 0.1
**0.15**	0.76	3.4 ± 0.1
**0.1**	0.79	3.5 ± 0.1
**0.05**	0.82	3.3 ± 0.3
**0.025**	0.86	2.7 ± 0.1
**0.015**	0.89	3.0 ± 0.1
**0.01**	0.90	3.0 ± 0.1
**0**	1.00	1.77 ± 0.09

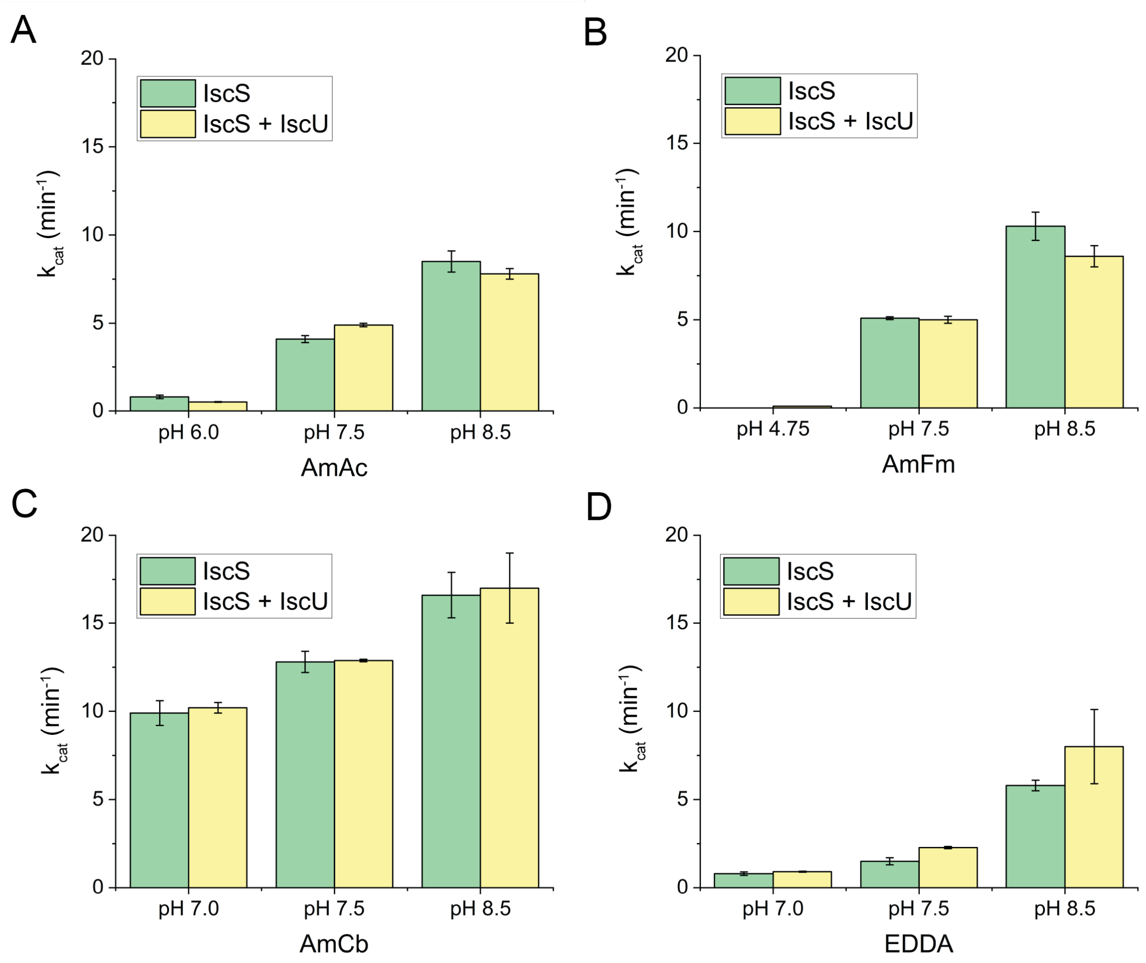

Figure S1. The rates of cysteine desulfurase activity of IscS ± IscU at 37°C were determined anaerobically using the sulfide detection assay in 200 mM AmAc (A), 200 mM AmFm (B), 200 mM AmCb (C), and 200 mM EDDA (D) at solution pH values near the upper and lower pKa of each buffer ion pair in addition to pH 7.5.

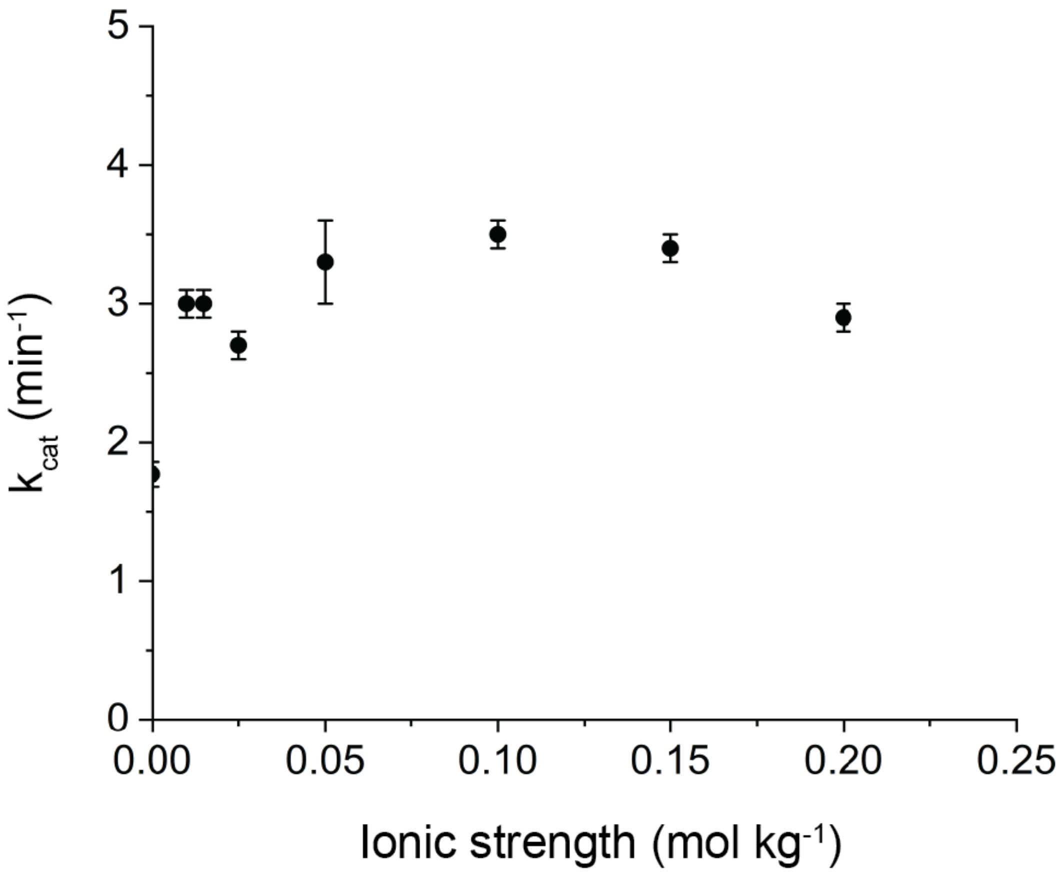

Figure S2. The rates of cysteine desulfurase activity of IscS were determined at 37°C using the sulfide detection assay in AmAc in decreasing concentrations to determine the effect of ionic strength on enzymatic efficiency.

Protein expression and purification

IscS was expressed in 6 L Luria-Bertani media in 1-L flasks with kanamycin (50 μg/mL). Cells were grown at 37°C to OD_{600} 0.6 and expression was induced with IPTG (0.5 mM). Growth was continued at 37°C for 5 h, and then the cells were harvested by centrifugation and stored at -80°C. Cells were resuspended in Ni A (50 mM Tris-HCl, 500 mM NaCl, 20 mM imidazole, 10% glycerol, pH 8.00) with 20 mg each lysozyme and protease inhibitor cocktail and lysed by sonication. IscS was loaded onto a HisTrap HP Ni-NTA column (Cytiva) with Ni A and eluted with Ni B (50 mM Tris-HCl, 500 mM NaCl, 500 mM imidazole, 10% glycerol, pH 8.0). Fractions containing IscS were pooled and placed in dialysis (50 mM HEPES, 300 mM NaCl, 5% glycerol, pH 8.0) overnight with TEV protease to cleave the N-terminal His-tag. After removing the cleaved tag from the mixture, IscS was loaded onto a HiPrep 26/60 Sephacryl S-300 size exclusion chromatography (Cytiva) equilibrated in S-300 buffer (50 mM HEPES, 250 mM NaCl, 5% glycerol, pH 8.0). Concentrated aliquots of the purified protein were stored at -80°C until use.

1. Kyte, J. D., R. F., A Simple Method for Displaying the Hydropathic Character of a Protein. *J. Mol. Biol.* **1982,** *157* (1), 105-132.
2. Ikai, A., Thermostability and Aliphatic Index of Globular Proteins. *J. Biochem.* **1980,** *88*, 1895-1898.
